# Estimating optimal sparseness of developmental gene networks using a semi-quantitative model

**DOI:** 10.1371/journal.pone.0176492

**Published:** 2017-04-21

**Authors:** Natsuhiro Ichinose, Tetsushi Yada, Hiroshi Wada

**Affiliations:** 1 Graduate School of Informatics, Kyoto University, Yoshida-Honmachi, Sakyo-ku, Kyoto, Japan; 2 Faculty of Computer Science and Systems Engineering, Kyushu Institute of Technology, Kawazu, Iizuka-shi, Fukuoka, Japan; 3 Graduate School of Life and Environmental Sciences, University of Tsukuba, Tennodai, Tsukuba, Japan; University of California, Davis, UNITED STATES

## Abstract

To estimate gene regulatory networks, it is important that we know the number of connections, or sparseness of the networks. It can be expected that the robustness to perturbations is one of the factors determining the sparseness. We reconstruct a semi-quantitative model of gene networks from gene expression data in embryonic development and detect the optimal sparseness against perturbations. The dense networks are robust to connection-removal perturbation, whereas the sparse networks are robust to misexpression perturbation. We show that there is an optimal sparseness that serves as a trade-off between these perturbations, in agreement with the optimal result of validation for testing data. These results suggest that the robustness to the two types of perturbations determines the sparseness of gene networks.

## Introduction

The purpose of this work is to clarify the mechanism determining the number of connections or sparseness of gene networks. Theoretically, even if each gene has full connections to all other genes, the desired expressions can be realized by the gene networks. Biologically, however, the gene networks are known to be sparsely connected [1]. It has been shown that robustness to expression noise is positively correlated with network modularity [2]. Since a high modularity implies low connections between modules, the networks can be maintained to be sparse in noisy environments. On the other hand, excessively sparse networks are obviously disadvantageous because a single *cis*-element mutation can cause gene dysfunction. Therefore, intermediate sparseness should be adopted by biological gene networks. In this work, we hypothesize that such an optimal sparseness is determined by maintaining robustness of the gene networks to perturbations. Undoubtedly, the biological function of the gene networks is also the main factor to determine their structure. However, the robustness to perturbations influences any gene in the gene networks, whereas a specific biological function may influence a subset of genes. Since the sparseness is a statistical quantity concerned with any gene, the robustness should play a major role in determining the sparseness.

For the robustness of the gene networks, we can consider two types of perturbations, mutational and environmental perturbations [3]. Mutational perturbation implies mutation of *cis*-elements and changes interactions of gene networks. Several theoretical works have shown that denser networks are more robust to mutational perturbations than sparser ones [4, 5]. On the other hand, environmental perturbation triggers gene expression noise or gene dysfunction. In this case, the robustness implies that an effect of a gene dysfunction does not spread into the other genes. In other studies such as epidemic spreading, in contrast to mutational perturbation, it has been shown that sparser networks are more robust to a dysfunction of a node than denser ones because an epidemic threshold is proportional to the inverse of the average number of connections [6]. In the gene networks, it is expected that the similar effect may be observed. In this work, we consider the connection-removal perturbation as mutational perturbation, and the misexpression perturbation as environmental perturbation. There should be an optimal sparseness of the networks that serves as a trade-off between these perturbations. We investigate whether this trade-off of the robustness to the two types of perturbations can predict the actual sparseness of the gene networks.

To analyze the effect of perturbations, a quantitative model to reproduce dynamics of biological gene networks is necessary. However, the expression data used in the network estimation are often qualitative binary data. In addition, temporal resolution of the expression data is intrinsically low because cell destruction is necessary in the measurement. To fulfill these required conditions, we adopt a semi-quantitative model on the basis of the Glass networks, which are represented by hybrid dynamical systems with real-valued nodes and logical interactions [7, 8]. Since several variables are represented by binary in the semi-quantitative model, binary expression data can be directly applied to the model. The binary variables also facilitate introducing time delay into the model. Since time-delay systems are infinite dimensional, their analysis is difficult in general. In our model, since the binary values are virtually transmitted through a delay, the system is finite dimensional. As results, interaction parameters are estimated by input and output values of the delay. Therefore, temporal resolution of the expression data is not necessarily required to be high. It should be noted that our model is represented by differential equations although binary variables and time delays are introduced into them. This implies that we can measure the effect of perturbations by the simulation of the differential equations.

Developmental gene networks are most appropriate for our purpose because the network dynamics directly influences the phenotype and is susceptible to the perturbations. In the landmark study of developmental gene networks, Peter *et al.* constructed a dynamic Boolean computational model from the expression data in the embryonic development of the sea urchin [9]. They showed that the model can predict the dynamics of the expressions. In this work, however, we propose a new method to estimate the gene networks without using their model, because of the following two reasons. One reason is the control of the number of connections in the estimation. Since our purpose is to analyze the sparseness as mentioned above, we require a method to control the number of connections, whereas that of their model is fixed. Another reason is the external signals. In other studies including that by Peter *et al.* [9], the external signals or the predefined expressions of genes are often assumed. Since our analysis is based on perturbations of the network dynamics, we attempt to construct more autonomous networks with self-determining intercellular signals than those used in the other studies.

We adopt the machine-learning method as the estimation method of the gene networks. In the machine learning, regularization is important for determining the sparseness of solutions. The LASSO (least absolute shrinkage and selection operator) method uses 1-norm of parameters as the regularization term [10]. It is known that sparse solutions can be obtained by the 1-norm regularization [11]. On the other hand, the ∞-norm regularization gives dense solutions similar to the 2-norm regularization used in support vector machine [12]. We combine the 1-norm and ∞-norm regularization methods and construct an adjustable sparse learning method that can control the sparseness.

## Materials and methods

### Semi-quantitative model of gene networks

**Fig 1:**
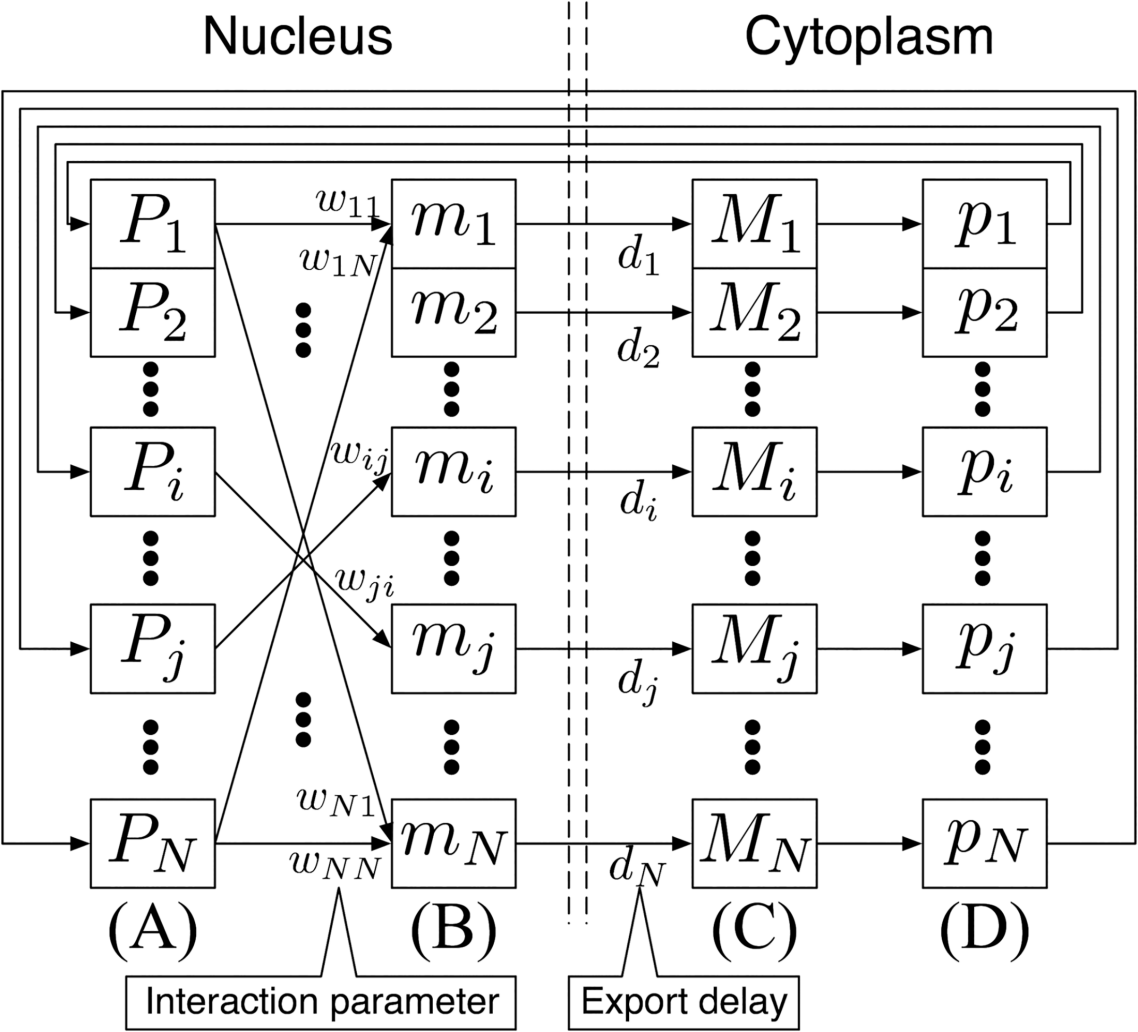
Model structure. Boxes show variables: (A) binary protein, (B) mRNA, (C) binary mRNA, and (D) protein. Arrows imply biological processes: (A)→(B) transcription, (B)→(C) mRNA export, (C)→(D) translation, and (D)→(A) protein import.

The model is represented by differential equations possessing continuous variables of mRNA and protein ((B) and (D) in Fig 1). However, to enable the network estimation by binary data, we introduce two types of binary variables into the model ((A) and (C) in Fig 1). “Semi-quantitative” is derived from these binary variables in the continuous differential equations. We explain the model on the basis of the biological processes in (A), (B), (C), and (D) in Fig 1.

**Transcription** (A)→(B): mRNA dynamics of the *i*-th gene is represented as follows,

$$m_{i}\left(t\right)=\sum_{j=1}^{N}w_{ij}P_{j}\left(t\right)+w_{i},$$ (1)

where *m*_*i*_ is the mRNA abundance in the nucleus, *t* is the continuous time, *N* is the number of genes, *w*_*ij*_ is the interaction parameter from the *j*-th gene to the *i*-th gene, and *w*_*i*_ is the basal level of transcription. Although the mRNA dynamics should be defined by a differential equation, we ignore the differential term because we assume that the generation and degradation of mRNAs proceed more rapidly than those of proteins. *P*_*j*_ is the binary variable of the protein imported from the cytoplasm into the nucleus, as explained later.

**mRNA export** (B)→(C): mRNA *m*_*i*_ is exported from the nucleus to the cytoplasm with an export delay and transformed into a binary variable. Then, the binary variable of mRNA *M*_*i*_ in the cytoplasm is determined by *m*_*i*_:

$$M_{i}\left(t\right)=\left\{\begin{array}{cc} \theta -\epsilon & \text{if}\;m_{i}\left(t-d_{i}\right)<\theta \\ \theta +\epsilon & \text{if}\;m_{i}\left(t-d_{i}\right)\geq \theta \end{array}\right.,$$ (2)

where *ϵ* is the coefficient of the effect on translation, *d*_*i*_ is the export delay from the nucleus to the cytoplasm, and *θ* is the threshold.

**Translation** (C)→(D): Protein dynamics of the *i*-th gene is represented by the following differential equation,

$$\tau \frac{dp_{i}\left(t\right)}{dt}=-p_{i}\left(t\right)+M_{i}\left(t\right),$$ (3)

where *p*_*i*_ is the protein abundance, and *τ* is the time constant of the protein.

**Protein import** (D)→(A): As mentioned above, protein *p*_*i*_ is imported from the cytoplasm into the nucleus and transformed into a binary variable. The binary variable of protein *P*_*i*_ in the nucleus is defined by,

$$P_{i}\left(t\right)=\left\{\begin{array}{cc} 0 & \text{if}\;p_{i}\left(t\right)<\theta^{\prime }\\ 1 & \text{if}\;p_{i}\left(t\right)\geq \theta^{\prime } \end{array}\right.,$$ (4)

where *θ*′ is the threshold. For simplicity, we assume that *θ*′ = *θ*. We also assume that the import delay of proteins is sufficiently smaller than the protein dynamics and can be ignored.

In our model, we assume that the export delays have a greater influence on transcriptional regulation than various other delays [13]. Whereas the number of delay parameters is *O*(*N*^2^) in general models [14], our model has only *N* delay parameters and we can easily estimate the delays on the basis of this assumption.

### Interpretation of binary expression data into model dynamics

The binary variables are used to interpret the binary expression data into the model dynamics. The binary mRNA variable *M*_*i*_(*t*) is the function of the binary protein variables *P*_*j*_(*t* − *d*_*i*_) for all *j* (see Eqs (1) and (2)). These properties enable us to determine the values of the interaction parameters from the binary expression data. However, we can obtain only the mRNA expression data, because it is difficult to detect protein abundance in general. Therefore, we express the protein variable by the mRNA variable approximately.

To express *P*_*j*_ by *M*_*j*_, we consider the delay *d* until which *p*_*j*_ reaches the threshold *θ* when *M*_*j*_ is inverted. Differential Eq (3) has two possible equilibria for each gene: $p_{j}^{*}=\theta \pm \epsilon$. Here we assume that *M*_*j*_ = *θ* − *ϵ* for a sufficiently long period and hence the gene is in an equilibrium *p*_*j*_(0) = *θ* − *ϵ* at *t* = 0. When *M*_*j*_ is inverted into *θ* + *ϵ* at *t* = 0, the delay *d* (*i.e.*, *p*_*j*_(*d*) = *θ*) is determined by solving differential Eq (3):

$$p_{j}\left(t\right)=\left(p_{j}\left(0\right)-M_{j}\left(t\right)\right)e^{-t/\tau }+M_{j}\left(t\right),$$ (5)

where we assume that *M*_*j*_(*t*) does not change in 0 < *t* ≤ *d*. At *t* = *d*, substituting *p*_*j*_(*d*) = *θ*, *p*_*j*_(0) = *θ* − *ϵ* and *M*_*j*_(*d*) = *θ* + *ϵ* into Eq (5), and solving it for *d*, we obtain

d=τlog2. (6)

The delay *d* has the same value even if *M*_*j*_ is inverted from *θ* + *ϵ* into *θ* − *ϵ*. Consequently, by using the quasi-steady-state approximation in which the time interval between adjacent inversions of *M*_*j*_ is sufficiently long, *P*_*j*_(*t*) is approximately determined by *M*_*j*_(*t* − *d*).

To be more specific, we convert *M*_*j*_ into $M_{j}^{\prime }$:

$$M_{j}^{\prime }\left(t\right)=\left\{\begin{array}{cc} 0 & \text{if}\;M_{j}\left(t\right)=\theta -\epsilon \\ 1 & \text{if}\;M_{j}\left(t\right)=\theta +\epsilon \end{array}\right..$$ (7)

Then, *P*_*j*_ is approximately represented as follows,

$$P_{j}\left(t\right)\approx M_{j}^{\prime }\left(t-d\right).$$ (8)

Therefore, from Eqs (1) and (2), the temporal relation of mRNAs is represented as follows,

$$M_{i}^{\prime }\left(t\right)\approx \{\begin{array}{cc} 0 & \text{if }\sum_{j=1}^{N}w_{ij}M_{j}^{\prime }\left(t-d_{i}-d\right)+w_{i}<\theta \\ 1 & \text{if }\sum_{j=1}^{N}w_{ij}M_{j}^{\prime }\left(t-d_{i}-d\right)+w_{i}\geq \theta \end{array}.$$ (9)

It is noted that Eq (9) has a form of linear classification in machine learning.

Let {*s*_1_(*t*), *s*_2_(*t*), …, *s*_*j*_(*t*), …, *s*_*N*_(*t*)} be the set of experimentally measured mRNA expression data: *s*_*j*_(*t*) ∈ {0, 1}. We assume that time *t* is discrete. The time discreteness of expression data does not hinder the estimation of parameter values in the continuous-time model. We can sufficiently estimate the interaction parameters from multiple data points with time interval *d*_*i*_ + *d*. Consequently, we can estimate the interaction parameters by applying machine learning to Eq (9) with $M_{j}^{\prime }=s_{j}$ for all *j*.

### Adjustable sparse learning

The gene networks are estimated by using the machine-learning method. The aim of our learning method is to estimate the networks having specified sparseness. It is known that sparse networks and dense networks can be obtained by the 1-norm regularization and the ∞-norm regularization, respectively. Combining the 1-norm and ∞-norm regularizations, we can control the sparseness of the estimated networks.

We construct the learning method as follows,

$$\text{Minimize}\;z_{i}\left(d_{i},\alpha ,C\right)=R\left(W_{i},\alpha \right)+C\sum_{k=1}^{K}\xi_{k},$$ (10)

subject to

$$\left(2s_{i}\left(k\right)-1\right)\left(\sum_{j=1}^{N}w_{ij}s_{j}\left(k-d_{i}-d\right)+w_{i}-\theta \right)\geq 1-\xi_{k},$$ (11)

$$w_{i}\geq 0,\;\xi_{k}\geq 0,\;i=1,2,\ldots ,N,\;k=1,2,\ldots ,K,$$

where *z*_*i*_ is the objective function, *α* is the sparseness parameter, *R* is the regularization term, *W*_*i*_ is the set consisting of *w*_*ij*_ for all *j* and *w*_*i*_, *C* is the soft-margin parameter, *K* is the length of time series, and *ξ*_*k*_ is the non-negative slack variable. *R* is defined as follows,

$$R\left(W_{i},\alpha \right)=\max\left(\alpha ||W_{i}{\left|\right|}_{\infty },\frac{\left|\right|W_{i}{\left|\right|}_{1}}{N+1}\right)$$ (12)

where || ⋅ ||_∞_ and || ⋅ ||_1_ are ∞-norm and 1-norm, respectively:

$$\left|\right|W_{i}{\left|\right|}_{\infty }=\max(|w_{i1}\left|,\right|w_{i2}\left|,\ldots ,\right|w_{iN}\left|,w_{i}\right),$$ (13)

and

$$\left|\right|W_{i}{\left|\right|}_{1}=\sum_{j=1}^{N}\left|w_{ij}\right|+w_{i}.$$ (14)

The temporal relation of mRNAs (Eq (9)) holds in the condition of Eq (11) if *ξ*_*k*_ = 0. The non-zero value of the slack variable (*ξ*_*k*_ > 0) implies that there are some errors in the expression data.

The sparseness parameter *α* in the regularization term *R* can control the sparseness of networks. If *α* = 0, *R* is equivalent to the 1-norm regularization:

$$R\left(W_{i},0\right)=\frac{\left|\right|W_{i}{\left|\right|}_{1}}{N+1}.$$ (15)

In this case, the method is equivalent to a standard sparse learning method and many interaction parameters become zero in the solution. On the other hand, if *α* = 1, *R* is equivalent to the ∞-norm regularization:

$$R\left(W_{i},1\right)=\left|\right|W_{i}{\left|\right|}_{\infty },$$ (16)

because the average (1-norm term) is less than or equal to the maximum (∞-norm term). In this case, all possible parameters have non-zero values. Therefore, we can obtain sparse networks when *α* is small, and dense networks when *α* is large.

Our learning method is similar to the elastic net regularization [15] which combines the 1-norm and 2-norm regularizations. The advantage of our method is that the learning method of Eqs (10) and (11) can be solved by linear programming, whereas the elastic net regularization requires quadratic programming. Therefore, the learning method can be applied to the estimation of large-scale networks.

The learning method does not directly estimate export delay *d*_*i*_. We explain the estimation method of export delay in S1 Appendix. We also explain the identification method of multiple optimal solutions in S1 Appendix.

### Expression data

We use the time series of expression data in the embryonic development of the sea urchin reported by Peter *et al.* [9]. The expression data include the activities of 40 genes which consist of 34 transcription factors, 5 signals, and 1 receptor. The gene expression is spatially and temporally specified. The spatial conditions are given by four embryonic domains: skeletogenic micromere, V2 mesoderm, V2 endoderm, and V1 endoderm. Each time series is measured at 1-h intervals until 30 h, except V2 mesoderm (until 16 h). All data are given by binary values.

**Fig 2:**
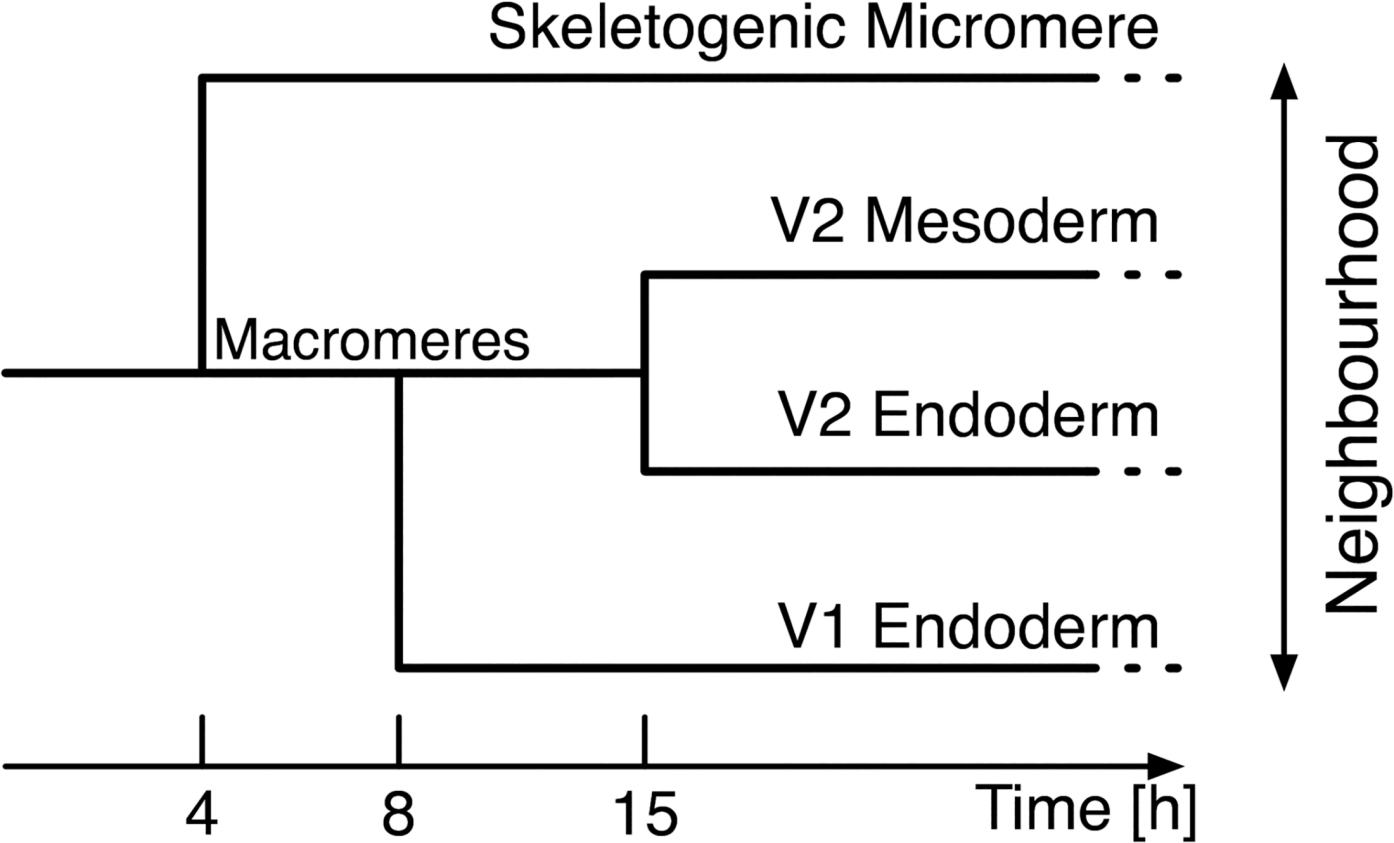
Time course of cell differentiation in the model. A signal can be transferred between only neighbouring domains.

In the simulation, we use the four equivalent networks corresponding to the four domains. We assume that a gene in a domain can receive signals from only neighbouring domains. The neighbourhood of domains varies with cell differentiation as shown in Fig 2. For example, since macromere differentiates into V1 and V2 at 8 h, V1 endoderm cannot receive signals from skeletogenic micromere after that time (Fig 2). In the simulation, the signal difference due to the cell differentiation produces the difference of the expression pattern in each domain.

We externally apply the reachability of signals between domains to the model. The reachability of signals depends on the physical distance between domains and the change of the physical distance is derived from cell proliferation. Since cell proliferation is not simulated in the model, the external application of the reachability of signals is necessary. It should be noted that the expressions of the signal genes themselves are autonomously determined by the network dynamics, whereas they are often given from an external source in other studies. Therefore, the intercellular signal exchange is autonomous except the effect of the reachability of signals. Unfortunately, however, there is no signal that stimulates the differentiation between micromere and macromere. To specify this differentiation, we externally apply the expression of *pmar1* gene to the gene networks as an exception. *pmar1* is known to control the specification of micromere [16].

Peter *et al.* have also used the results of perturbation experiments [9, 17] in which the activity of each of the 23 regulatory genes is interrupted and the effects on 191 genes in total are observed (data from http://sugp.caltech.edu/endomes/qpcr.html). We use this data set to evaluate the sparseness parameters *α* and the soft-margin parameter *C*.

### Statistics

We use F-measure *F* to evaluate the estimated networks:

$$F=\frac{2SN\cdot PPV}{SN+PPV},$$ (17)

where *SN* is the sensitivity:

$$SN=\frac{TP}{TP+FN},$$ (18)

and *PPV* is the positive predictive value:

$$PPV=\frac{TP}{TP+FP}.$$ (19)

*TP*, *FN*, and *FP* are the numbers of true positives, false negatives, and false positives, respectively. F-measure is a harmonic average of SN and PPV, and higher F-measure implies more accurate estimation. True negative is not directly used in evaluation. This is because the number of positive examples is fewer than that of negative examples in our data set. In such a case, F-measure gives a fair evaluation. Since the definitions of *TP*, *FN*, and *FP* are dependent on each analysis, we explain them in each section.

## Results and discussion

### Estimation of developmental gene networks

We can obtain multiple optimal solutions by means of the learning method. However, each optimal solution of a gene is independent of that of any other gene. This is because the variation of connections belonging to the gene has no effect on the other genes if the gene shows the correct expression. In the simulation, we identify 1,000 optimal solutions of the whole system. To construct each of the optimal solutions, we perform the following procedure: We randomly and independently choose one from among the 100 optimal solutions of each gene (with repetition). By concatenating the chosen solutions of all genes, we construct an optimal solution of the whole system.

**Fig 3:**
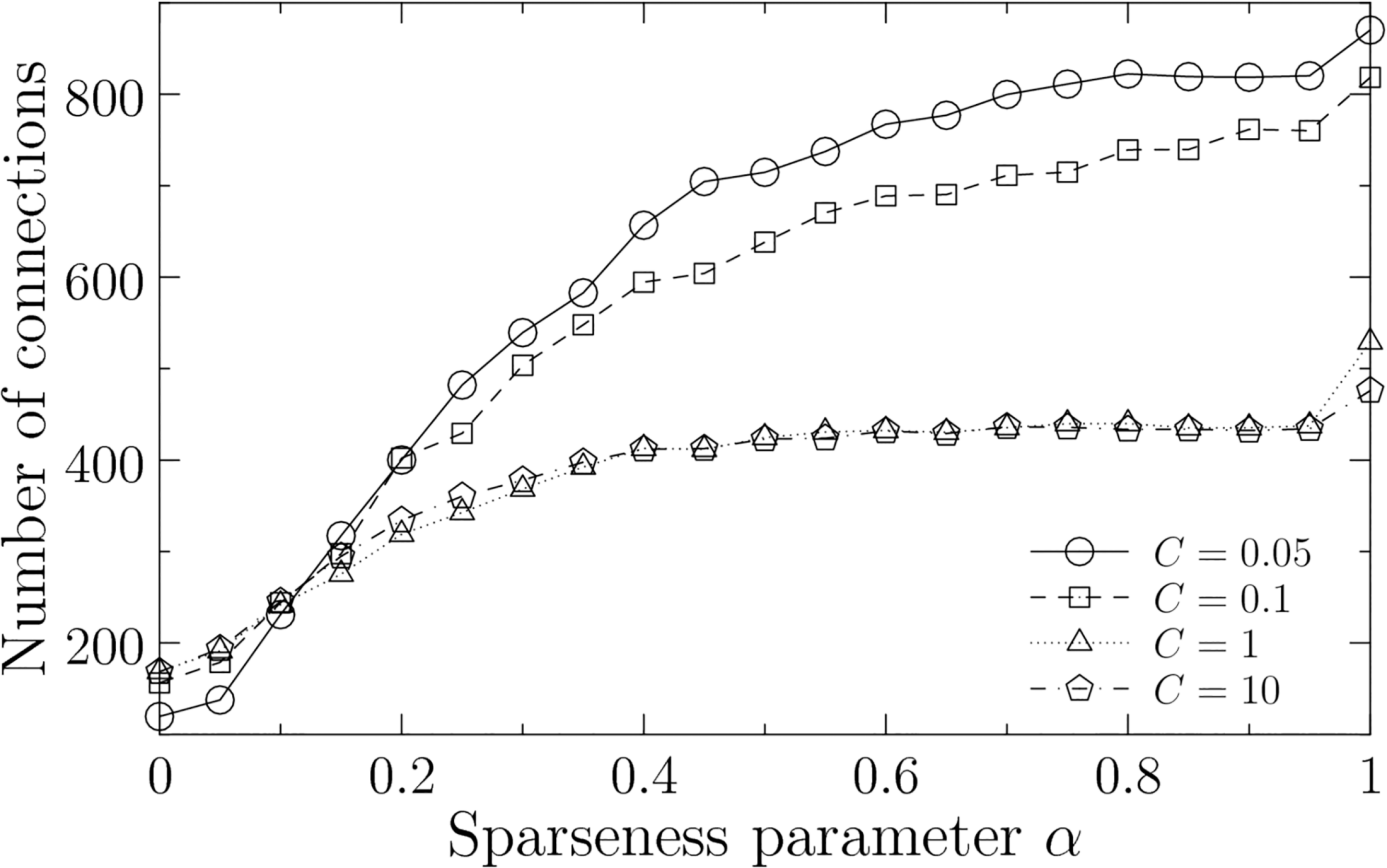
Number of estimated connections as a function of sparseness parameter *α*. We show the average number for each soft-margin parameter *C* in 1,000 optimal solutions. The other parameters are fixed at *d* = 2 [h], *d*_*min*_ = 0 [h], *d*_*max*_ = 2 [h], and *θ* = 1.

As shown in Fig 3, we can control the sparseness of the estimated networks by the sparseness parameter *α*. Although the sparseness is also dependent on soft-margin parameter *C*, it is saturated for large *C* (*C* = 1 and *C* = 10 in Fig 3). In general, whereas the optimal value of *C* depends on the actual ratio of errors included in the experimental data, that of *α* depends on the actual number of connections.

From the simulation of the estimated networks, we derive the estimated expression data. We use the same initial state of Peter *et al.* [9]: Maternal mRNAs are expressed at an early stage (*ets1* until 10 h, *mat-n_b-cat* and *otx-alpha* until 6 h, and *tbr* and *tel* until 9 h) and the activities of the other genes are repressed until 6 h. Since the system is defined in continuous time, we measure *M*_*i*_ for all *i* in all domains at intervals of 1 h to compare them with the experimental data. To evaluate accuracy of the estimated expression data, we use F-measure. In this analysis, we define TP by the number that an expression time of the estimated data agrees with that of the experimental data in a same domain. FP and FN are the numbers of the estimated data and the experimental data subtracted by TP, respectively.

**Fig 4:**
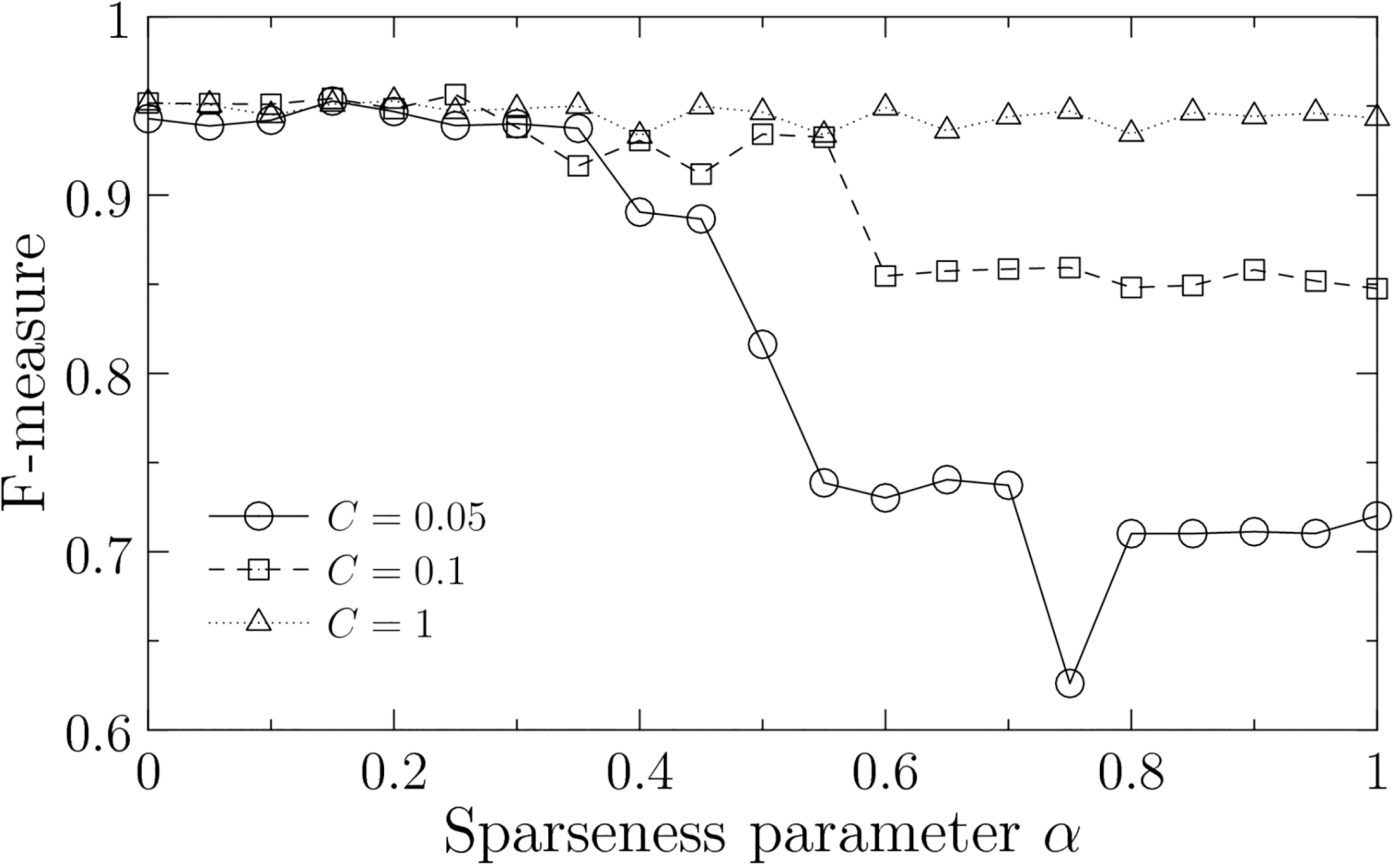
Accuracy for time-series data as a function of sparseness parameter *α*. We show the average F-measure for each soft-margin parameter *C* in 1,000 optimal solutions. F-measure is calculated in the experimental data after 6 h. The other parameters are fixed at *d* = 2 [h], *d*_*min*_ = 0 [h], *d*_*max*_ = 2 [h], *ϵ* = 0.5, and *θ* = 1.

As shown in Fig 4, the estimated networks have high F-measure for each parameter setting at *α* less than 0.5. These results suggest that our method is effective for estimating gene networks. In the following results, we use the parameter range 0 ≤ *α* ≤ 0.5.

**Fig 5:**
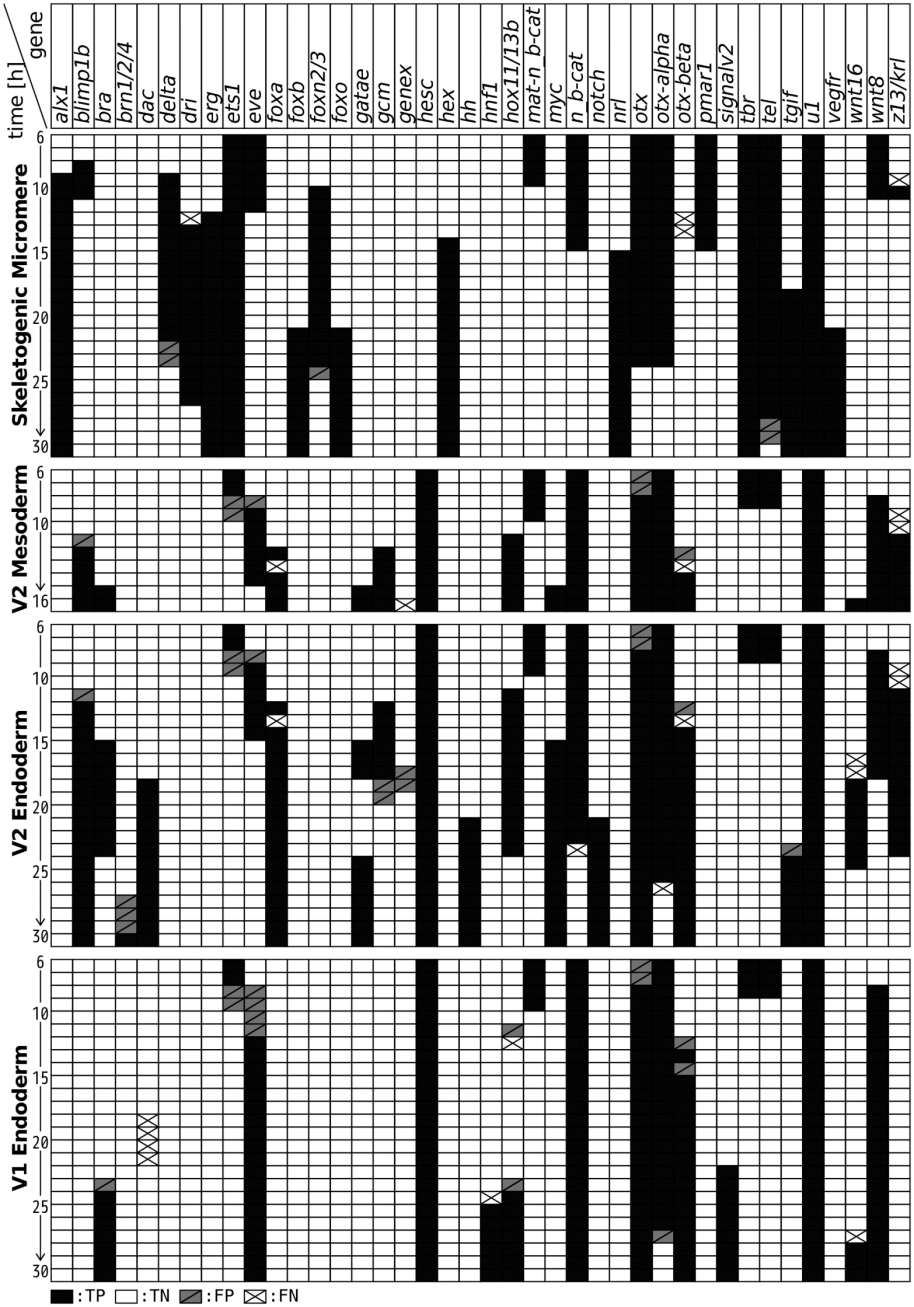
Estimated time series of mRNA expressions. These time series are examples at *α* = 0.25 and *C* = 0.1, and we use the first optimal solution. Compared with the experimental data, each expression is classified into True Positive (TP), True Negative (TN), False Positive (FP), or False Negative (FN). The other parameters are fixed at *d* = 2 [h], *d*_*min*_ = 0 [h], *d*_*max*_ = 2 [h], *ϵ* = 0.5, and *θ* = 1.

Fig 5 shows an example of the estimated time series of *M*_*i*_ for all *i*. It should be noted that the false expressions mainly occur around the times of true positives. Since our model is defined in continuous time, temporal difference can occur due to discrete sampling. In addition, the actual time resolution is 3 h in the experimental data [9]. Therefore, we can ignore the errors before and after consecutive expression. These results indicate that the estimated networks have the ability to reproduce the experimental data with high accuracy.

It should be also noted that the time series of the estimated networks is measured by simply solving the differential equations. In such a case, an error of gene expression affects the network dynamics after its occurrence and is accumulated in time. Nevertheless, the estimated networks show temporally accurate dynamics. This implies that the gene networks are adequately reconstructed.

In the following results, we use the set of the estimated networks calculated here. Although we perform perturbation analysis in the following sections, it does not mean that we recalculate the estimated networks for perturbed time series.

### Validation of optimal sparseness

To validate the optimal sparseness, we evaluate the sparseness parameter *α* and the soft-margin parameter *C* by using the testing data that are independent of the time-series data used in learning. As the testing data, we use the data of the gene perturbation experiments [9, 17]. In the experiments, the effects on each gene are measured by whether the number of transcripts is significantly increased or decreased by a perturbed gene at several time intervals. In other words, the testing data are gene expression changes between the situations in which a gene perturbation is present or absent. Therefore, the type of the testing data are not similar to that of the learning data that are gene expression itself. However, we can apply an emulated gene perturbation to the estimated networks and obtain gene expression changes without difficulty, because the estimated networks are represented by dynamical systems.

In the gene perturbation experiments, the gene expression changes are measured in four time intervals [12*h* − 16*h*], [17*h* − 22*h*], [23*h* − 25*h*], and [26*h* − 30*h*]. In the simulation, we adopt expression changes that occur most frequently in each time interval. Unfortunately, the spatial domains are not separated to measure gene expression in the experiments. Then, we assume that the gene expression experimentally measured is averaged in all spatial domains. In the simulation, therefore, we obtain the gene expression change as the average in all domain. We define TP by the number that a gene expression change (including increase or decrease) of the estimated data agrees with that of the experimental data in each time interval. FP and FN are the numbers of the estimated data and the experimental data subtracted by TP, respectively.

**Fig 6:**
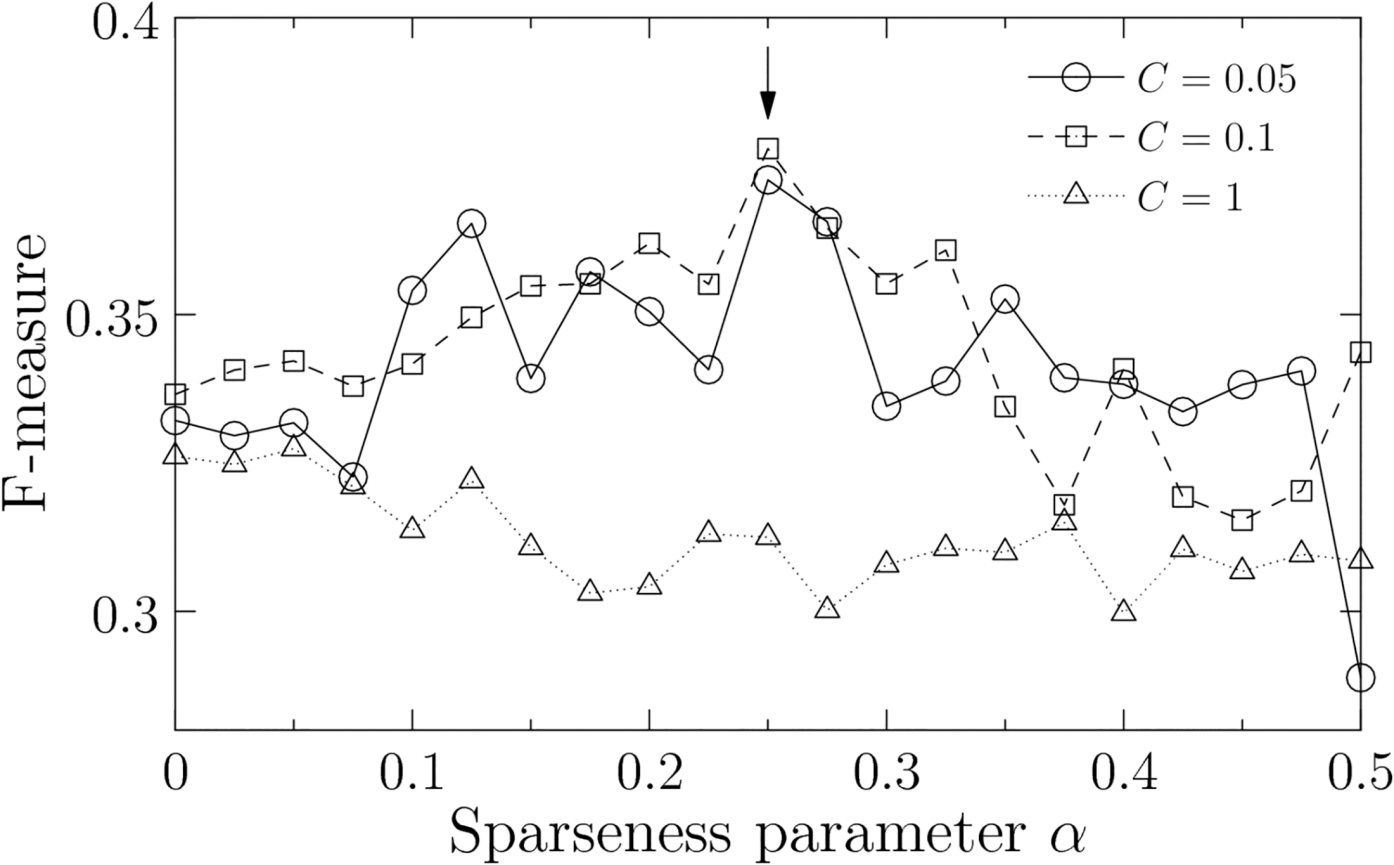
Accuracy for gene perturbation data as a function of sparseness parameter *α*. We show the average F-measure for each soft-margin parameter *C* in 1,000 optimal solutions. The optimal F-measure is observed at (*α*, *C*) = (0.25, 0.1) (indicated by an arrow). The other parameters are fixed at *d* = 2 [h], *d*_*min*_ = 0 [h], *d*_*max*_ = 2 [h], *ϵ* = 0.5, and *θ* = 1.

In Fig 6, we show accuracy of the estimated data by F-measure. Unfortunately, the accuracy is low in comparison with that of the learning data (Fig 4). However, this is due to the difference of the types of data. In the previous section, we could evaluate the learning data by a direct comparison between the estimated and experimental data because the time series of expression data was obtained in each spatial domain. In the testing data, however, such a direct comparison is hampered by the facts that the experimental data are *changes* of gene expression and are not separated into the spatial domains. Since we cannot identify the spatial domain in which the changes of gene expression occur, the reproduction of a time series resulted by a gene perturbation is not feasible and hence we cannot directly compare the time series of the estimated data with that of the experimental data. On the other hand, we can show that the estimated and experimental data are significantly consistent with each other by the test of independence (Pearson’s *χ*^2^-test: *χ*^2^ = 333, *p*-value< 0.01 at (*α*, *C*) = (0.25, 0.1)). Therefore, we consider that the estimated networks can sufficiently capture the feature of the gene perturbation experiments.

Consequently, we estimate that the optimal sparseness is observed at (*α*, *C*) = (0.25, 0.1) that corresponds to the best F-measure in Fig 6. In this case, the estimated networks have 429 connections out of the 1,560 possible connections in average (Fig 3). This result indicates that the best estimated network is not necessarily the sparsest one (e.g., 120 connections at *α* = 0 and *C* = 0.05). We discuss about the process to determine the sparseness of the gene networks in the next section.

### Two types of perturbations determine sparseness

To evaluate the robustness of the networks, we apply the connection-removal and misexpression perturbations to the estimated networks. We define that the estimated networks are robust if the dynamics change by perturbations is small. Therefore, we define TP by the number that an expression time of the perturbed networks agrees with that of the unperturbed networks in a same spatial domain. FP and FN are the numbers of gene expressions in the perturbed and unperturbed networks subtracted by TP, respectively. Then, higher F-measure implies more robust networks.

**Fig 7:**
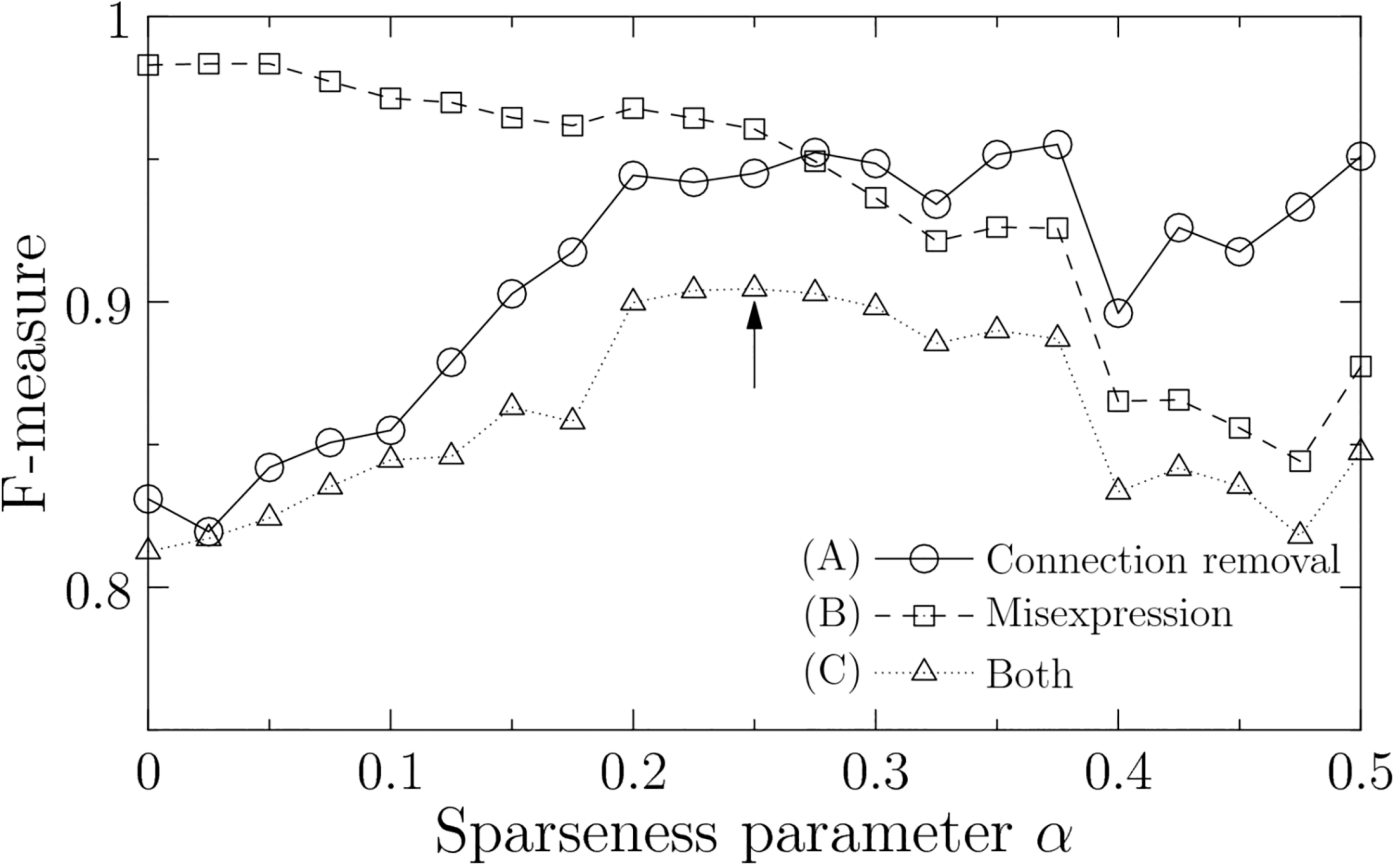
Robustness of perturbed networks as a function of sparseness parameter *α*. As the robustness, we show the average F-measure in 1,000 optimal solutions. The types of perturbations are (A) connection-removal perturbation (*n*_*c*_ = 10), (B) misexpression perturbation (*λ* = 10, *μ* = 0.01), and (C) the addition of both perturbations. Optimal robustness in (C) is observed at *α* = 0.25 (indicated by an arrow). The other parameters are fixed at *C* = 0.1, *d* = 2 [h], *d*_*min*_ = 0 [h], *d*_*max*_ = 2 [h], *ϵ* = 0.5, and *θ* = 1.

The connection-removal perturbation corresponds to the mutation of *cis*-elements. We assume that the *n*_*c*_ *cis*-elements are simultaneously mutated and lose their function, *i.e.*, the *n*_*c*_ non-zero weights *w*_*ij*_ are converted into *w*_*ij*_ = 0. Under the condition of the constant number of connection removals, the robustness is positively correlated with the sparseness parameter *α* (Fig 7(A)). These results suggest that the dense networks have high robustness to connection-removal perturbation. This is expected because the effect of connection removals is small since a gene in the dense networks has many *cis*-elements [4, 5].

The misexpression perturbation corresponds to the gene dysfunction and the open failure of chromatin. The misexpression perturbation includes both under- and over-expression. To emulate the misexpression perturbation, it is necessary that the noise maintains its effect on expression changes for a certain period of time. We adopt the random walk noise because it has a long time correlation. We apply the random walk noise to *m*_*i*_ as follows,

$$m_{i}\left(t\right)=\sum_{j=1}^{N}w_{ij}P_{j}\left(t\right)+w_{i}+\int_{0}^{t}\sum_{k=1}^{\infty }\mu_{k}\delta \left(t^{\prime }-t_{k}\right)dt^{\prime },$$ (20)

where *μ*_*k*_ is randomly chosen from ±*μ*, *δ* is the delta function, and the time interval (*t*_*k*_ − *t*_*k*−1_) is randomly determined by the exponential distribution:

$$P\left(t_{k}-t_{k-1}\right)=\lambda e^{-\lambda (t_{k}-t_{k-1})},$$ (21)

where *λ* is the intensity parameter of the noise. Since the average time interval of noise is 1/*λ*, a high value of *λ* implies high density of noise. Under the condition of constant noise, the robustness is negatively correlated with the sparseness parameter *α* (Fig 7(B)). These results suggest that the sparse networks have high robustness to the misexpression perturbation. This result is unexpected because dense networks are considered to be more robust to noise than sparse ones in general. This discrepancy is derived from the random walk noise whose effect is not instant but maintained for a certain period. This property of the misexpression perturbation is responsible for the cascading failure that occurs in power transmission, computer networking, and finance [18]. In the cascading failure, failure of a single node can trigger successive failures at the system level. Since a gene affects many other genes in the dense networks and the cascading failure is enhanced by that, dense networks have lower robustness than sparse ones as a result.

**Fig 8:**
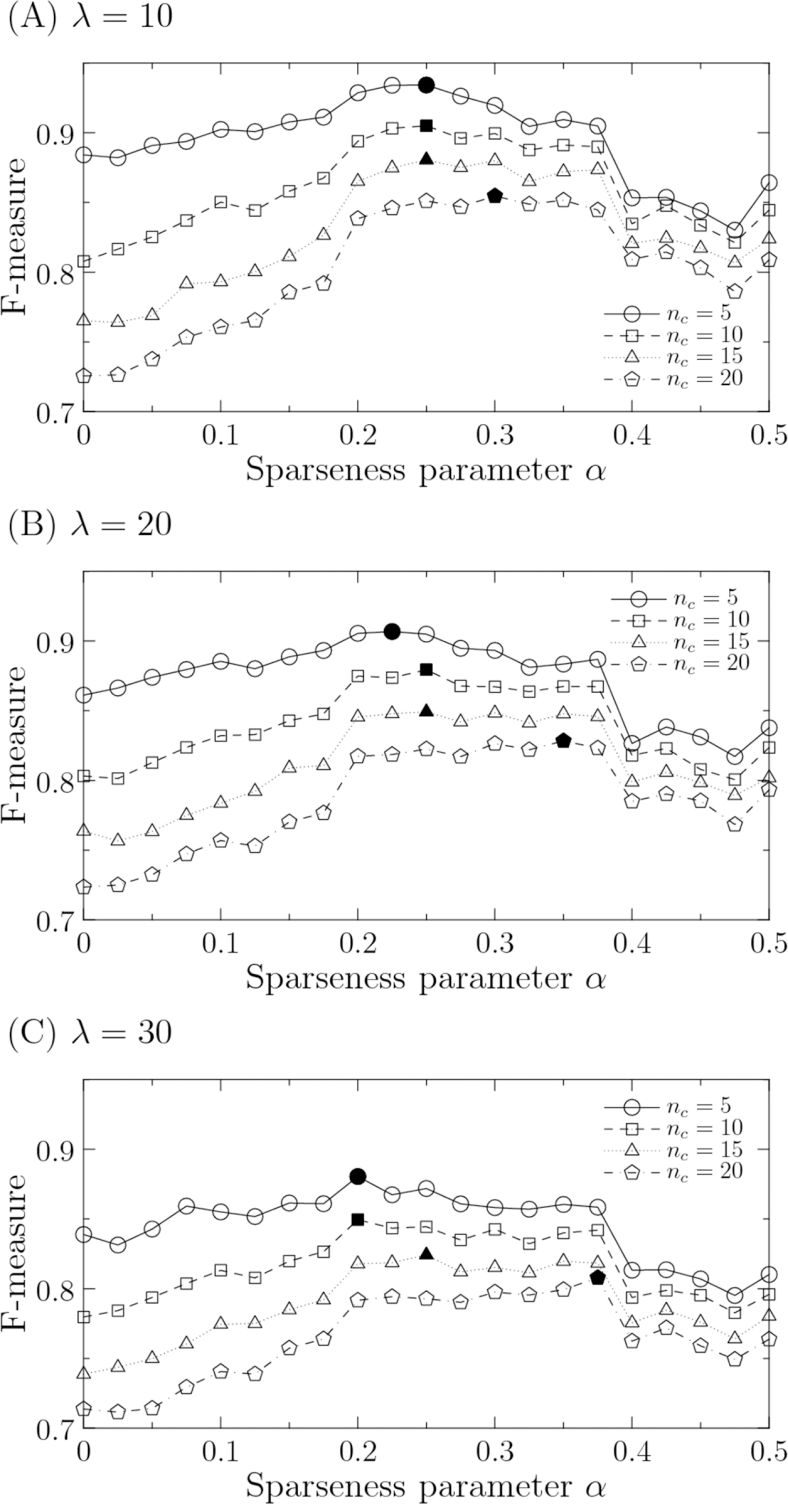
Robustness of perturbed networks as a function of sparseness parameter *α* at various noise intensities (A) *λ* = 10, (B) *λ* = 20, and (C) *λ* = 30. Both connection-removal and misexpression perturbations are applied to the estimated networks. As the robustness, we show the average F-measure for each number of connection removals *n*_*c*_ in 1,000 optimal solutions. The filled mark indicates the maximum value in each profile. The other parameters are fixed at *μ* = 0.01, *C* = 0.1, *d* = 2 [h], *d*_*min*_ = 0 [h], *d*_*max*_ = 2 [h], *ϵ* = 0.5, and *θ* = 1.

The connection-removal perturbation occurs mutationally or evolutionarily, whereas the misexpression perturbation is due to environmental noise. Thus, the time scales of the connection-removal and misexpression perturbations differ from each other. However, we assume that the gene networks are constantly exposed to the misexpression perturbation because it is environmental noise. As results, the gene networks that have evolutionarily received the connection-removal perturbation are also exposed to the misexpression perturbation in the same way. In actual situations, therefore, it is expected that both connection-removal and misexpression perturbations occur for a certain (possibly evolutionary) period of time. We show the results when we simultaneously apply both of the perturbations to the estimated networks in Fig 7(C). The sparseness parameter *α* has a trade-off point because the profiles of the connection-removal and misexpression perturbations are contrary to each other. The optimal value of the robustness is obtained at *α* = 0.25 and this value is equivalent to the optimal value of the validation results (*α* = 0.25 in Fig 6). In Fig 8, we assess the robustness for various values of perturbation parameters in both connection-removal and misexpression perturbations. Although the variation is relatively high at *n*_*c*_ = 20, the optimal value of *α* is almost invariant.

It should be noted that this analysis (Fig 7) is independent of that of the previous section (Fig 6). In the previous section, we estimated the optimal sparseness of actual gene networks by using the experimental perturbation data. In this case, the estimated networks are optimal if they have the same sparseness of the actual gene networks. In this section, we evaluate the robustness to the perturbations without considering what is the actual sparseness. In this case, the estimated networks are optimal if their behaviour does not change by the perturbations. It is important that the optimal solutions of both results are consistent with each other. These results suggest that the optimal sparseness is determined by maintaining the robustness of the gene networks to the connection-removal and misexpression perturbations that have the contrastive characteristics to each other.

## Conclusion

We have proposed a semi-quantitative model of gene networks and its adjustable sparse learning method. We have shown that the sparseness has the optimal value determined by maintaining the robustness to the connection-removal and misexpression perturbations using the estimated networks from the experimental expression data. In this work, since we investigated only sparseness of specific developmental gene networks, the result is restrictive. Therefore, further work is required to clarify the generality of optimal sparseness, theoretically and biologically.

## Supporting information

S1 Appendix Supplemental explanations of learning method. Estimation of export delay, and identification of multiple optimal solutions.
(PDF)
