# Supplementary material for: Estimating optimal sparseness of developmental gene networks using a semi-quantitative model
Source: PLoS One. 2017 Apr 21;12(4):e0176492. doi: 10.1371/journal.pone.0176492 (PMC5400252; doi:10.1371/journal.pone.0176492)
Supplement: S1 Appendix — Estimation of export delay, and identification of multiple optimal solutions. (PDF) [file pone.0176492.s001.pdf]

Supporting Information for: Estimating Optimal Sparseness of Developmental Gene Networks Using a Semi-quantitative Model

Natsuhiro Ichinose[1], Tetsushi Yada[2], Hiroshi Wada[3]

1 Graduate School of Informatics, Kyoto University, Yoshida-Honmachi, Sakyo-ku, Kyoto 606-8501, Japan
2 Faculty of Computer Science and Systems Engineering, Kyushu Institute of Technology, 680-4 Kawazu, Iizuka-shi, Fukuoka 820-8502, Japan
3 Graduate School of Life and Environmental Sciences, University of Tsukuba, Tennodai, Tsukuba 305-8672, Japan

Estimation of export delay

The learning method of Eqs. (10) and (11) in the main text does not directly estimate export delay d_i. We assume that d_i is discrete because the expression data are discrete in time. We also assume that d_i is bounded: $d_i \in [d_{min}, d_{max}]$. Note that the delay parameter is dependent on only i in condition (11). This implies that we can estimate the optimal parameters of the i-th gene by changing only d_i and keeping the other delays constant, whereas the combinatorial optimization is necessary in general models. Then, we can estimate d_i with the following optimal solution,

$$z_i^*(\alpha, C) = \min_{d_i \in [d_{min}, d_{max}], W_i} z_i(d_i, \alpha, C). \tag{S1}$$

Identification of multiple optimal solutions

Suboptimal solutions are often useful to analyze the property of the estimated networks. After estimating the optimal network by the learning method, we obtain the suboptimal solutions by iterating the following method,

$$\text{Minimize } z_i'(d_i^*, \alpha, C) = R(W_i', \alpha) + C \sum_{k=1}^{K} \xi_k, \tag{S2}$$

subject to Eq. (11) and,

$$z_i(d_i^*, \alpha, C) \le z_i^*(\alpha, C) + \nu, \tag{S3}$$

where,

$$W_i' = \{c_{i1}w_{i1}, c_{i2}w_{i2}, \dots, c_{ij}w_{ij}, \dots, c_{iN}w_{iN}, w_i\}, \tag{S4}$$

d_i^* is the optimal delay determined by Eq. (S1), and ν is the control parameter of suboptimality (to be explained later). c_{ij} is the restriction coefficient of w_{ij} and is changed by the iteration: c_{ij} is initially 1, but is increased by 1 if w_{ij} is non-zero in the previous iteration. This implies that it is difficult for the non-zero connection in the previous iteration to become non-zero in this iteration, and we can obtain a different solution from the previous one.

In addition to condition (11), we use condition (S3) that restricts the solutions. Note that the left side of Eq. (S3) is equal to the original objective function Eq. (10). Then, we can obtain the ν-nearest solutions of the optimal solution z_i^*. Empirically, we can obtain several solutions even if $\nu = 0$, *i.e.*, there exist multiple optimal solutions. We use the multiple optimal solutions by fixing this parameter at $\nu = 0$ for all results.
